# Supplementary material for: Control of telomerase recruitment and end protection by independent shelterin components
Source: Nat Commun. 2026 Jan 15;17:1733. doi: 10.1038/s41467-026-68433-0 (PMC12913867; doi:10.1038/s41467-026-68433-0)
Supplement: Supplementary file 1 — Supplementary Information [file 41467_2026_68433_MOESM1_ESM.pdf]

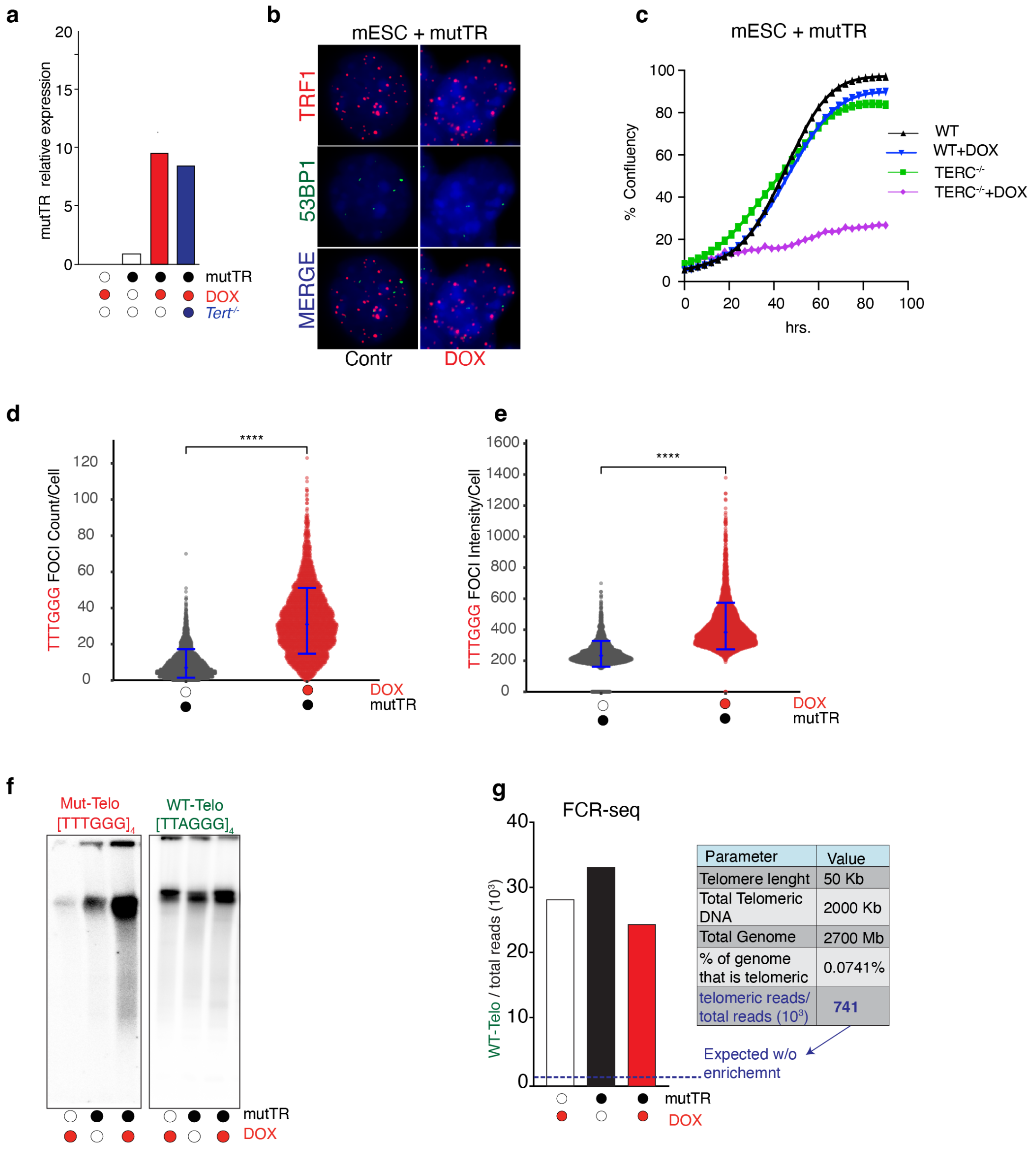

Supplementary Figure 1. Characterization of mutTR expression and mutant repeat incorporation.

a RT-qPCR quantification of mutTR expression in the absence or presence of doxycycline (DOX) in cells with or without integration of the mutTR construct. **b** Representative immunofluorescence-FISH (IF-FISH) images showing 53BP1 (green) and TRF1 (red) localization in cells with or without mutTR induction for 64 hours. (**c**) Cell proliferation of Wild type mESCs compared to TERC knockout mESCs upon induction of mutTR expression by doxycycline treatment (+DOX) or control conditions. Proliferation was monitored by confluency using the IncuCyte S3 system. (**d**) Plot representing automated TTTGGG foci count per nucleus. Each dot represents one cell; with a minimum of 2500 cells scored per condition. Two-tailed unpaired *t*-test; **** indicates P < 0.0001. (E) Plot representing automated quantitative analysis TTTGGG intensity per cell . Each dot represents one cell; with a minimum of 2500 cells scored per condition. Two-tailed unpaired *t*-test; **** indicates P < 0.0001 **(f)** Telomeric restriction fragment (TRF) analysis of cells with or without mutTR expression following 64 hours of DOX treatment. Denaturing in-gel hybridization was performed using a probe specific for mutant telomeric repeats (right panel); the same blot was stripped and re-probed for wild-type telomeric repeats. (**d**) Quantification of telomeric sequencing reads per million (RPM) of total aligned reads following telomere enrichment. The dotted line indicates the expected RPM of telomeric reads without enrichment, calculated based on the genomic proportion of telomeric DNA.

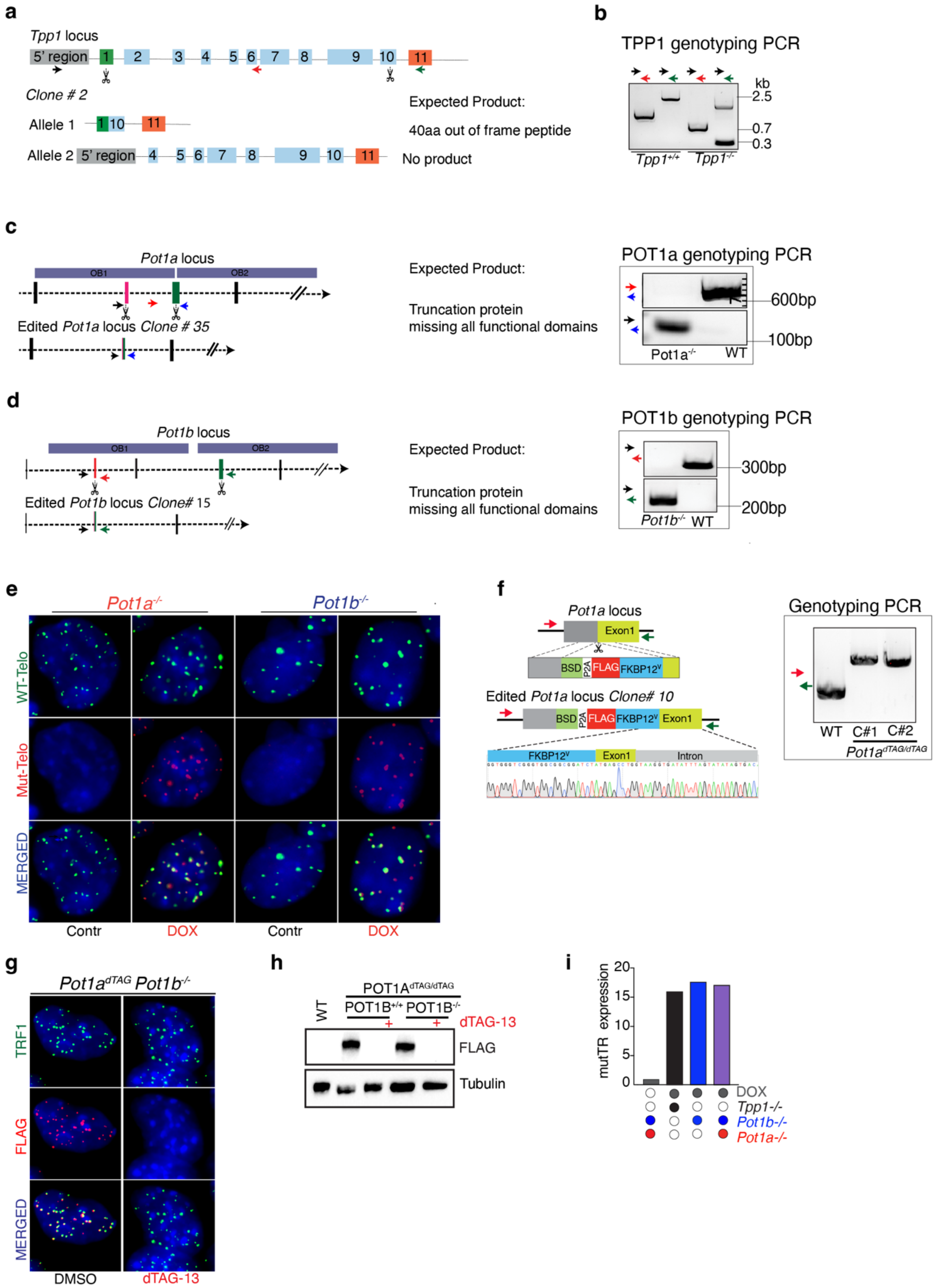

Supplementary Figure 2. Generation and validation of TPP1 and POT1 mutant alleles.

(**a**) Schematic representation of the *Tpp1/Acd* genomic locus showing gene structure and the predicted CRISPR-Cas9 cut site (scissors). (**b**) Genotyping PCR across the CRISPR-Cas9 cut site comparing wild-type (WT) and *Tpp1*$^{-/-}$ clones. Primer positions are indicated on the schematic in (a). (**c**) Schematic of the *Pot1a* genomic locus showing gene structure, the CRISPR-Cas9 target site, and relevant protein domains.

(**d**) Schematic of the *Pot1b* genomic locus, CRISPR-Cas9 cut site, and protein domains. Genotyping PCR verifies allele disruption; primer positions are indicated on the locus map. (**e**) Representative FISH images showing mESCs of the indicated genotypes hybridized with PNA probes for wild-type ([TTAGGG], green) and mutant ([TTTGGG], red) telomeric repeats following 64 hours of mutTR induction with DOX. (**f**) Schematic of the *Pot1a* genomic locus and the knock-in repair template used to generate the endogenous 3×FLAG-FKBP12^{F36V}-POT1a fusion allele. The template includes homology arms, a blasticidin resistance gene (BSD), a P2A peptide, a 3×FLAG tag, and the FKBP12^{F36V} degron. The CRISPR-Cas9 cut site used for integration is marked (scissors). Sanger sequencing confirms the correct junction; genotyping PCR confirms biallelic targeting. (**g**) Immunofluorescence staining for TRF1 (green) and FLAG (red) in mESCs expressing 3×FLAG-FKBP12^{F36V}-POT1a from the endogenous *Pot1a* locus (*Pot1a^{dTAG} Pot1b$^{-/-}$*), treated with DMSO or dTAG-13. **(h)** Western blot of mESCs expressing 3×FLAG-FKBP12^{F36V}-POT1a treated with or without dTAG-13 (500 nM, 5 hours). (**i**) RT-qPCR quantification of mutTR expression in the absence or presence of DOX in cells of the indicated genotypes.

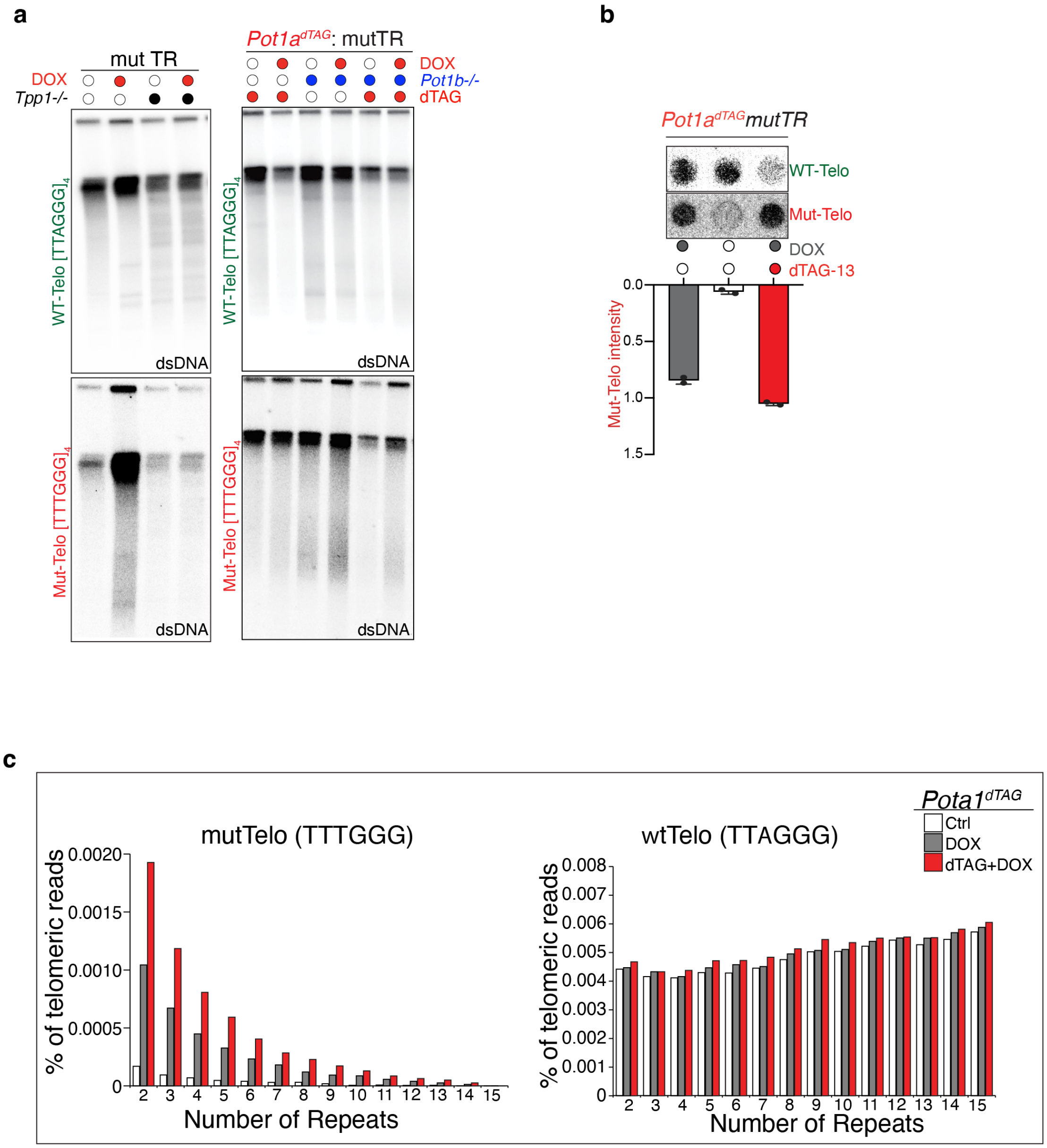

Supplementary Figure 3. TRF analysis in mutant backgrounds.

(**a**) Telomeric restriction fragment (TRF) analysis of genomic DNA isolated from cells of the indicated genotypes. When indicated, cells were treated with doxycycline (DOX) for 64 hours to induce the expression of mutTR and/or with dTAG-13 for 70 hours to induce depletion of POT1a. Denaturing in-gel hybridization was performed using a probe specific for mutant telomeric repeats (upper panel); the same blot was subsequently stripped and re-probed for wild-type repeats. (**b**) Genomic DNA isolated from *Pot1a*^{dTAG} cells, either untreated, treated with doxycycline (DOX) or treated with dTAG-13 (dTAG). DNA was hybridized with probes complementary to wild-type [TTAGGG] or mutant [TTTGGG] telomeric repeats. The graph shows the ratio of signal intensity between the mutant and wild-type telomeric probes for each condition. Data represent mean from two independent experiments.. **(c)** Fraction of reads containing the indicated number of repeats based on FCR-seq analysis of genomic DNA from cells that were untreated (white), treated with DOX (gray bars), or treated with both dTAG-13 and DOX (red bars). The left graph shows the distribution of mutant telomeric repeats; the right graph shows the distribution of wild-type telomeric repeats.

a

Parental cell genotype	Target genotype	+ve clones / total
WT	*Pot1a*$^{-/-}$	7 / 24
WT	*Pot1b*$^{-/-}$	10/24
WT	*Tpp1*$^{-/-}$	2 /18
Pot1a$^{-/-}$	*Pot1b*$^{-/-}$	**0 /50**

b

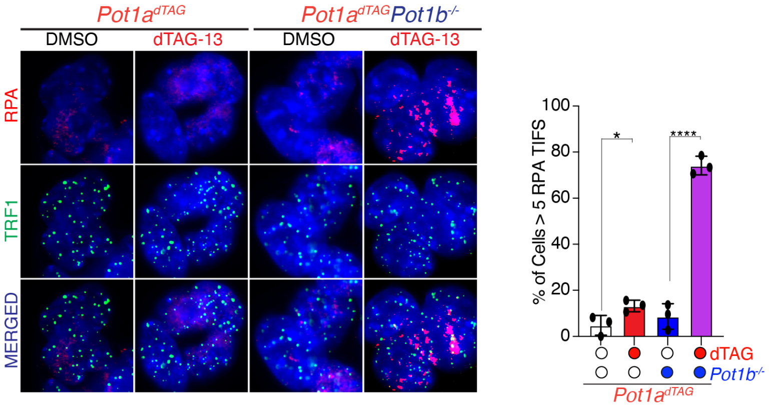

c

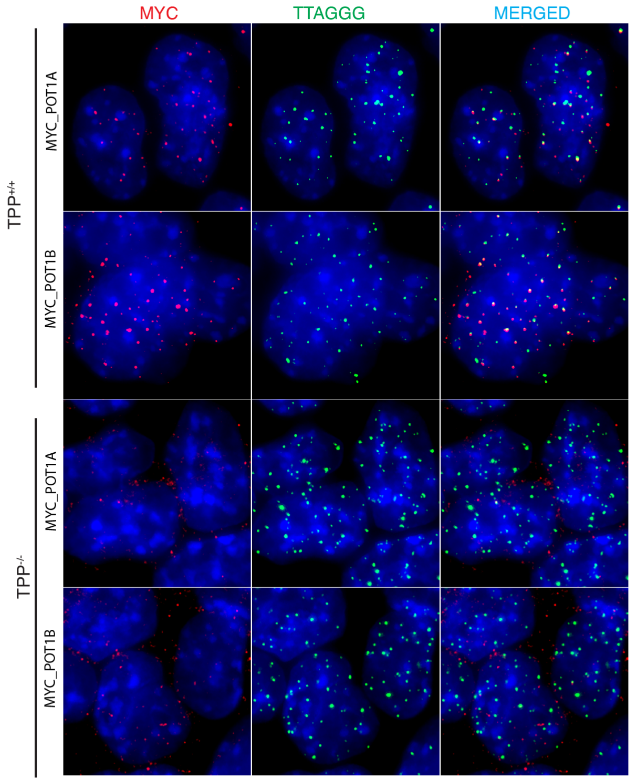

d

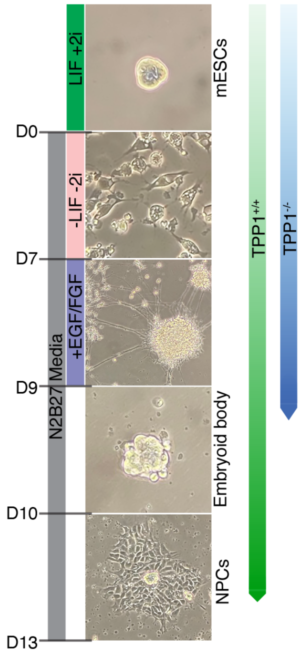

Supplementary Figure 4. Gene editing efficiency and DNA damage response at telomeres. (**a**) Table summarizing the number of positive clones obtained versus the total number screened after CRISPR-Cas9 targeting for each genotype. (**b**) Representative immunofluorescence images showing RPA2 (red) and TRF1 (green) localization in the indicated genotypes. Where indicated, dTAG-13 was used to induce degradation of POT1a-FKBP12^{F36V}. The graph shows quantification of cells with ≥5 telomere dysfunction-induced foci (TIFs). Data represent mean ± s.d. from three biological replicates. Statistical analysis was performed using a two-tailed unpaired *t*-test; (* P =0.043; **** P < 0.0001). **(c)** Representative

IF-FISH images showing the localization of ectopically expressed MYC-tagged POT1a and POT1b in TPP1-proficient and TPP1-deficient cells. MYC-tagged POT1a and POT1b were detected using an anti-MYC antibody (red), and telomeres were visualized using a [TTAGGG] PNA probe (green) **(d)** Schematic overview and representative images illustrating the generation of embryoid bodies (EBs) and their differentiation into neural progenitor cells (NPCs). TPP1-proficient cells successfully progress to the differentiated NPC stage, whereas TPP1-deficient cells fail to advance beyond the EB stage.

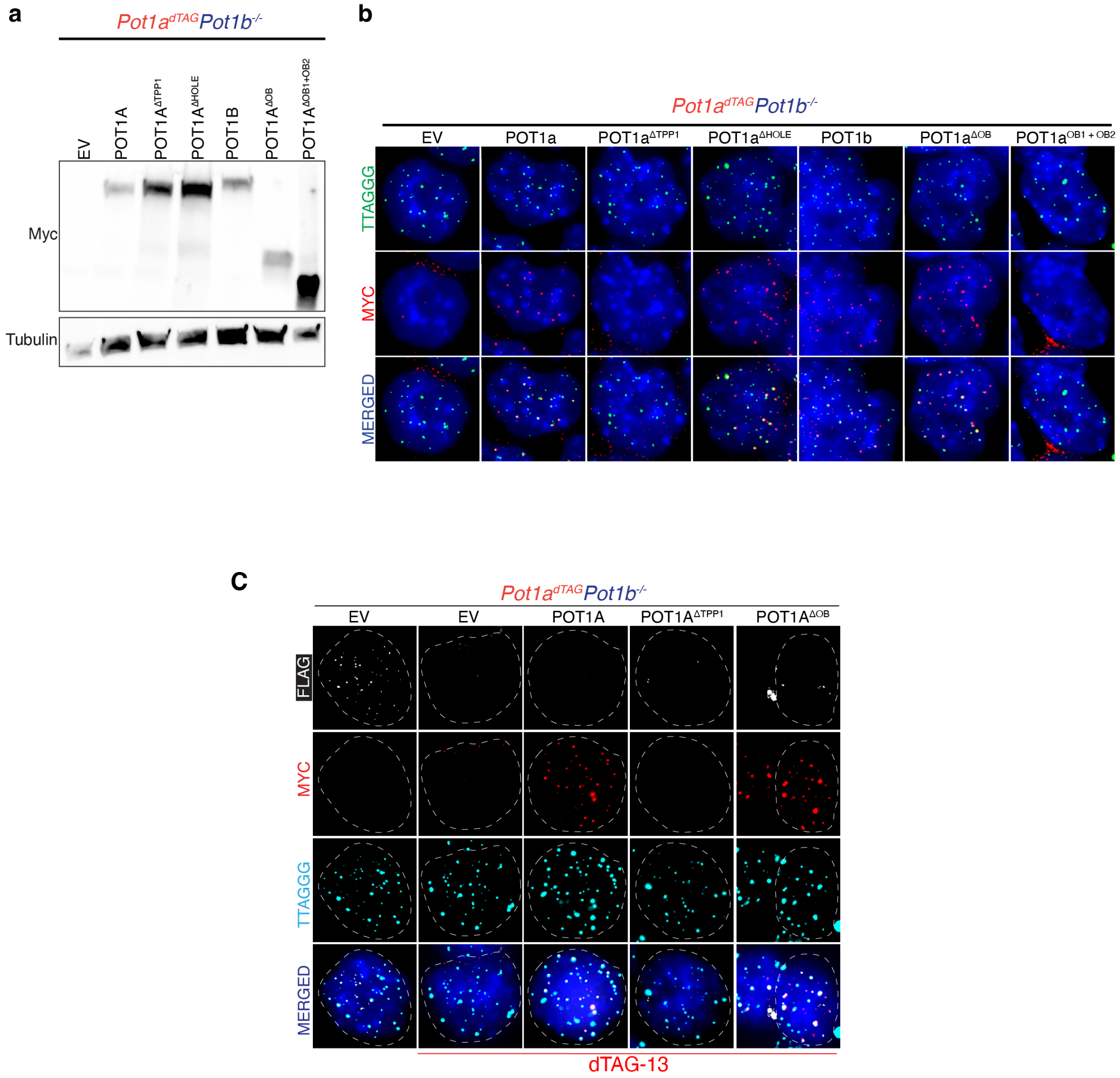

Supplementary Figure 5. POT1a localization.

(**a**) Western blot showing ectopic expression of MYC-tagged POT1a variants and POT1b in POT1a^{dTAG} Pot1b$^{-/-}$ cells. Tubulin was used as loading control. (**b**) Representative IF-FISH images showing localization of ectopically expressed MYC-tagged POT1a variants and POT1b in POT1a^{dTAG} Pot1b$^{-/-}$ cells. MYC-tagged POT1a and POT1b were detected using an anti-MYC antibody (red), and telomeres were visualized using a [TTAGGG] PNA probe (green). **(c)** Representative IF-FISH images showing localization of ectopically expressed MYC-tagged POT1a variants in POT1a^{dTAG} Pot1b$^{-/-}$ cells treated with dTAG-13. Endogenous POT1a-FKBP12^{F36V} was detected by anti-FLAG staining (white); ectopic variants were detected with anti-MYC antibody (red). Telomeres were visualized with a [TTAGGG] PNA probe (cyan).

A

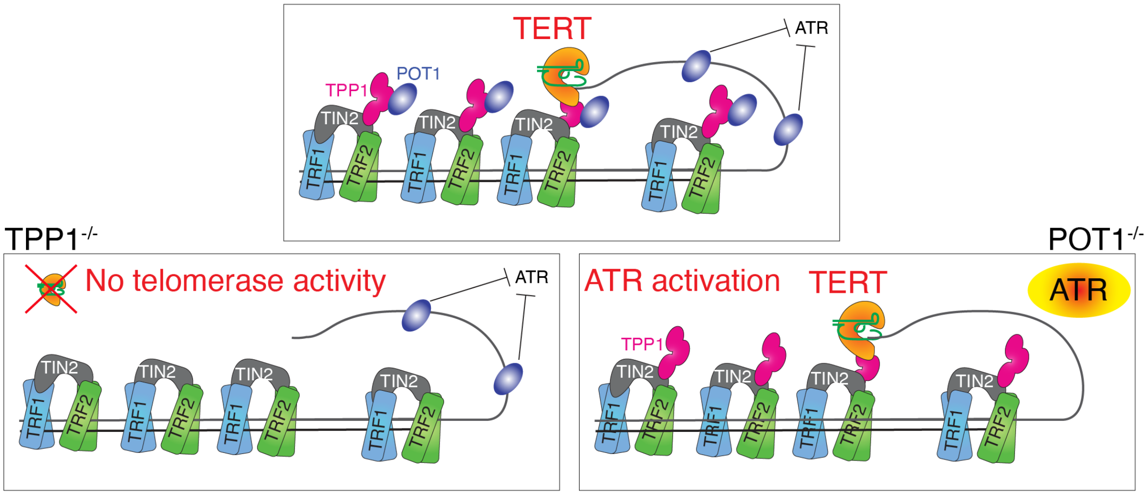

Supplementary Figure 6. Working model

Model for TPP1-POT1 mediated telomere maintenance. TPP1 recruit's telomerase to telomeres in a POT1-independent manner. While POT1 loading is facilitated by TPP1, in conditions of sufficient expression, POT1 can independently bind the single-stranded overhang via its OB domains and suppress ATR activation. In this model, telomerase-mediated extension and POT1-dependent end protection occur concurrently, without requiring a dynamic "open-closed" telomere state. Regulation may occur through modulation of the TPP1-TIN2 or TPP1-POT1 interaction by post-translational modification or an unknown factor that displaces POT1 from the telomeric end.
