# Supplementary material for: Control of telomerase recruitment and end protection by independent shelterin components
Source: Nat Commun. 2026 Jan 15;17:1733. doi: 10.1038/s41467-026-68433-0 (PMC12913867; doi:10.1038/s41467-026-68433-0)
Supplement: Supplementary file 2 — Reporting Summary [file 41467_2026_68433_MOESM2_ESM.pdf]

nature portfolio

Corresponding author(s): Eros Lazzerini Denchi

Last updated by author(s): Dec 22, 2025

Reporting Summary

Nature Portfolio wishes to improve the reproducibility of the work that we publish. This form provides structure for consistency and transparency in reporting. For further information on Nature Portfolio policies, see our Editorial Policies and the Editorial Policy Checklist.

Statistics

For all statistical analyses, confirm that the following items are present in the figure legend, table legend, main text, or Methods section.

n/a	Confirmed	
☐	☒	The exact sample size (n) for each experimental group/condition, given as a discrete number and unit of measurement
☐	☒	A statement on whether measurements were taken from distinct samples or whether the same sample was measured repeatedly
☐	☒	The statistical test(s) used AND whether they are one- or two-sided<br>*Only common tests should be described solely by name; describe more complex techniques in the Methods section.*
☐	☒	A description of all covariates tested
☐	☒	A description of any assumptions or corrections, such as tests of normality and adjustment for multiple comparisons
☐	☒	A full description of the statistical parameters including central tendency (e.g. means) or other basic estimates (e.g. regression coefficient) AND variation (e.g. standard deviation) or associated estimates of uncertainty (e.g. confidence intervals)
☐	☒	For null hypothesis testing, the test statistic (e.g. F, t, r) with confidence intervals, effect sizes, degrees of freedom and P value noted<br>*Give P values as exact values whenever suitable.*
☒	☐	For Bayesian analysis, information on the choice of priors and Markov chain Monte Carlo settings
☒	☐	For hierarchical and complex designs, identification of the appropriate level for tests and full reporting of outcomes
☒	☐	Estimates of effect sizes (e.g. Cohen's d, Pearson's r), indicating how they were calculated

Our web collection on statistics for biologists contains articles on many of the points above.

Software and code

Policy information about availability of computer code

Data collection	Zeiss Zen software (Zen Blue) was used to acquire IF, IF-FISH images. Bio-Rad ChemiDox Imager was used to acquire images of the gels and blots. BioRad CFX Maestro Instrument and software were used for RT-qPCR experiments.
Data analysis	The analysis of sequencing data was performed with R packages: parallel/stringr/dplyr/tidyr/tools

For manuscripts utilizing custom algorithms or software that are central to the research but not yet described in published literature, software must be made available to editors and reviewers. We strongly encourage code deposition in a community repository (e.g. GitHub). See the Nature Portfolio guidelines for submitting code & software for further information.

Data

Policy information about availability of data

All manuscripts must include a data availability statement. This statement should provide the following information, where applicable:

- Accession codes, unique identifiers, or web links for publicly available datasets
- A description of any restrictions on data availability
- For clinical datasets or third party data, please ensure that the statement adheres to our policy

ll data supporting the findings of this study are available within the paper and its Supplementary Information. All raw and processed data will be made available upon request (by mail at eros. lazzerinidenchi@nih.gov). Sequencing data will be deposited into the GEO database before pubblication.

Research involving human participants, their data, or biological material

Policy information about studies with human participants or human data. See also policy information about sex, gender (identity/presentation), and sexual orientation and race, ethnicity and racism.

Reporting on sex and gender	N/A
Reporting on race, ethnicity, or other socially relevant groupings	N/A
Population characteristics	N/A
Recruitment	N/A
Ethics oversight	N/A

Note that full information on the approval of the study protocol must also be provided in the manuscript.

Field-specific reporting

Please select the one below that is the best fit for your research. If you are not sure, read the appropriate sections before making your selection.

☒ Life sciences ☐ Behavioural & social sciences ☐ Ecological, evolutionary & environmental sciences

For a reference copy of the document with all sections, see nature.com/documents/nr-reporting-summary-flat.pdf

Life sciences study design

All studies must disclose on these points even when the disclosure is negative.

Sample size	For experiments involving quantification of positive cells (e.g. TIFs positive) or chromosome fusions n=3 was chosen as the minimal replicate number, and sample size was determined by the number of positive cells within the replicates. We determined this to be sufficient owing to internal control (specific staining of positionally defined cell types using known markers) and low observed variability between stained samples.
Data exclusions	Data were not excluded from analysis.
Replication	All replication attempts were successful and observed data were consistent with orthogonal methods and previously known results.
Randomization	Cells and chromosomes used for imaging were selected randomly and analyzed equally with no sub-sampling and thus, there was no requirement for randomization.
Blinding	Investigators were blinded to group allocation during data collection and analysis, to minimize bias.

Reporting for specific materials, systems and methods

We require information from authors about some types of materials, experimental systems and methods used in many studies. Here, indicate whether each material, system or method listed is relevant to your study. If you are not sure if a list item applies to your research, read the appropriate section before selecting a response.

Materials & experimental systems

n/a	Involved in the study	
☐	☒	Antibodies
☐	☒	Eukaryotic cell lines
☒	☐	Palaeontology and archaeology
☒	☐	Animals and other organisms
☒	☐	Clinical data
☒	☐	Dual use research of concern
☒	☐	Plants

Methods

n/a	Involved in the study	
☒	☐	ChIP-seq
☒	☐	Flow cytometry
☒	☐	MRI-based neuroimaging

Antibodies

Antibodies used	FLAG -M2 FLAG mouse SIGMA-F1804

Antibodies used	FLAG FLAG rabbit Cell signaling-14793S<br>cMYC-9E10 MYC mouse Santa Cruz-Sc-40<br>MYC MYC mouse Cell signaling-2276<br>53BP1 53BP1 rabbit Novus-hB100304<br>TRF1 TRF1 rat abcam-ab192629<br>RPA RPA2 rabbit Invirtogen-PA1-23299<br>TUBULIN Tubulin mouse Millipore Sigma-T5168
Validation	Well established commercial antibodies. In this study negative and positive controls have been included to verify the specificity of staining

Eukaryotic cell lines

Policy information about cell lines and Sex and Gender in Research

Cell line source(s)	Mouse Embryonic stem cells (mESCs) (https://www.atcc.org/products/crl-1821)
Authentication	Staining for the pluripotency marker (OCT4) was used to define the differentiation status of the cell lines used.
Mycoplasma contamination	In the lab cell lines are routinely inspected by fluorescence and using commercial kits to detect mycoplasma. In this study we did not detect any mycoplasma contamination.
Commonly misidentified lines (See ICLAC register)	No commonly misidentified cell lines were used.

Plants

Seed stocks	*Report on the source of all seed stocks or other plant material used. If applicable, state the seed stock centre and catalogue number. If plant specimens were collected from the field, describe the collection location, date and sampling procedures.*
Novel plant genotypes	*Describe the methods by which all novel plant genotypes were produced. This includes those generated by transgenic approaches, gene editing, chemical/radiation-based mutagenesis and hybridization. For transgenic lines, describe the transformation method, the number of independent lines analyzed and the generation upon which experiments were performed. For gene-edited lines, describe the editor used, the endogenous sequence targeted for editing, the targeting guide RNA sequence (if applicable) and how the editor was applied.*
Authentication	*Describe any authentication procedures for each seed stock used or novel genotype generated. Describe any experiments used to assess the effect of a mutation and, where applicable, how potential secondary effects (e.g. second site T-DNA insertions, mosiacism, off-target gene editing) were examined.*
